# ESCORTing proteins directly from whole cell-lysate for single-molecule studies

**DOI:** 10.1016/j.ab.2017.07.022

**Published:** 2017-10-15

**Authors:** Shwetha Srinivasan, Jagadish P. Hazra, Gayathri S. Singaraju, Debadutta Deb, Sabyasachi Rakshit

**Affiliations:** a Department of Chemical Sciences, Indian Institute of Science Education and Research, Mohali, Punjab, 140306, India; b Centre for Protein Science Design and Engineering, Indian Institute of Science Education and Research, Mohali, Punjab, 140306, India

**Keywords:** Surface modification, Protein-protein interactions, Pull-down assays

## Abstract

We have developed a method for Enzymatic Sortase-assisted Covalent Orientation-specific Restraint Tethering (ESCORT) recombinant proteins onto surfaces directly from cell-lysate. With an improved surface passivation method, we obviate the cumbersome purification steps even for single molecule studies that demand high purity in the sample. We demonstrated high-specificity of the method, high-passivity of the surface and uncompromised functional integrity of anchored proteins using single molecule fluorescence and force-mapping. We anticipate that this method will substantially reduce the investment by way of time, money and energy in the area of single molecule studies.

## Introduction

Interaction of proteins with biological and chemical molecules plays an important role in various cellular biosynthetic and degradation pathways controlling major processes in living organisms [1]. Quantitative understanding of these interactions is of primary importance to gain insight into different basic biochemical processes happening inside a living organism.

Over the time a number of ensemble techniques like bead based bioassays, surface plasmon resonance, isothermal calorimetry, differential scanning calorimetry, microfluidics and single molecule techniques like fluorescence based assays, force-based assays have been developed to characterize protein-protein, protein-DNA, protein-small molecule interactions quantitatively [2], [3]. Most of these techniques require homogeneous immobilization of protein onto a solid support in a specific orientation along with maintaining its functionality. Special attention towards surface preparation is therefore essential, specifically for single molecule techniques where nonspecific interactions due to surface artifacts or non-specifically bound molecules can impair the population completely [4] [5]. The major steps involved in surface preparation are: surface-passivation [6] [7], firm attachment of molecules [8] [9] preferably with covalent binding [10] [11], at proper distributions and specific orientations and reproducibility. Molecules in the purest form is another essential requirement for reproducibility in single molecule measurements and this involves multistep purifications.

Here we report a method of surface passivation and subsequently enzymatic sortase-assisted covalent orientation specific restraint tether (ESCORT)ing of proteins from the whole cell-lysates appropriate for single molecule experiments. Sortase-A enzyme from *Staphylococcus aureus* has been employed to catalyze the direct immobilization of recombinant protein bearing small genetically encoded peptide tag (LPXTG) onto surfaces pre-attached with glycine [12]. This method supersedes the cumbersome manifold purification process and efficiently attaches target proteins directly from cell-lysate with as little as nM expression.

## Results and discussion

### Homogeneous distribution of target molecules on the surface for single molecule study

**Fig. 1:**
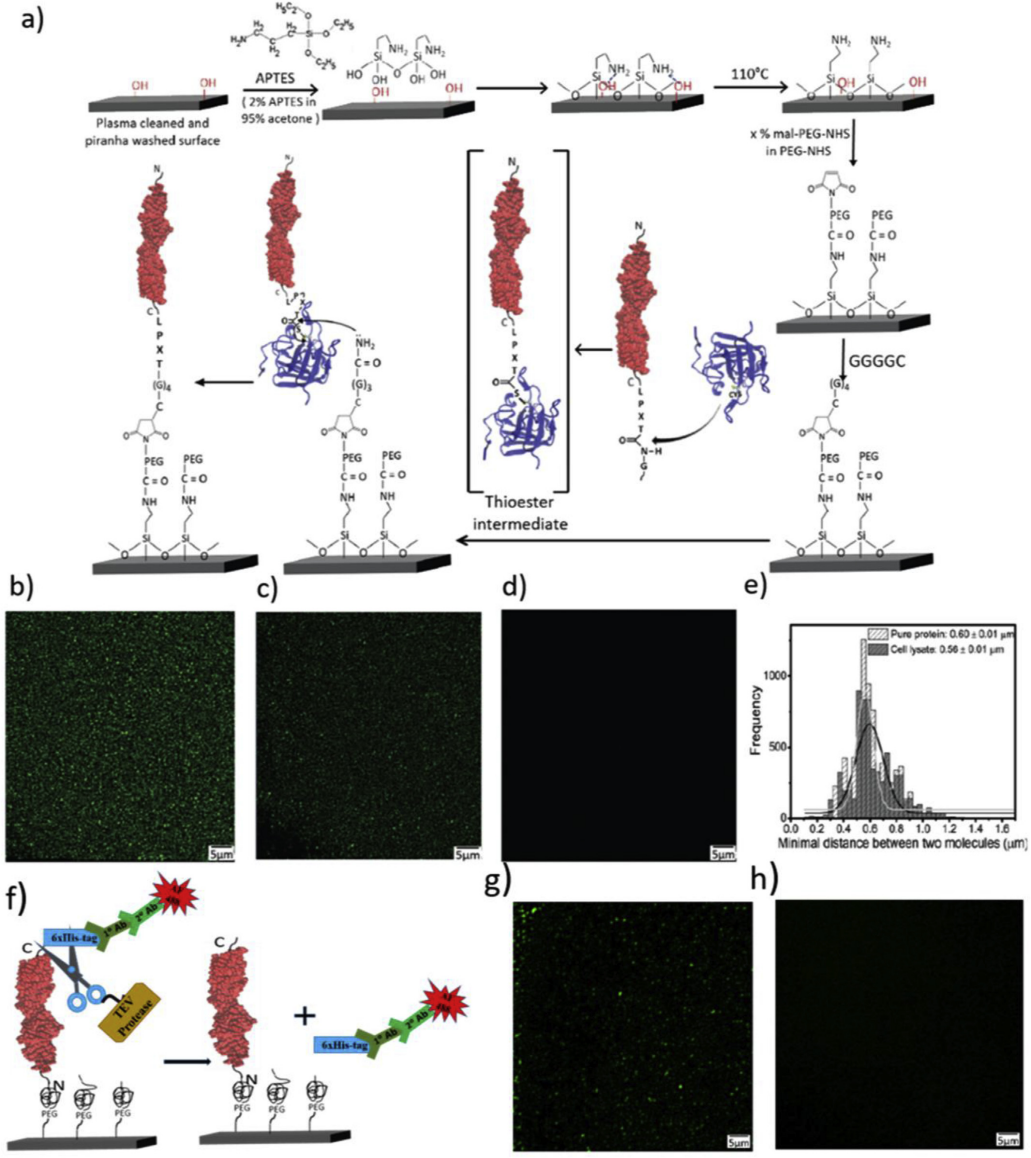
ESCORTing of proteins. (a) Reaction scheme of making highly passive surface and ESCORTing. TIRF images of Cdh23 ESCORTed from (b) affinity and chromatography pure fraction and (c) crude cell-extract directly on 10% mal-PEG-NHS modified glass-coverslip. (d) Fluorescence image of modified-coverslip incubated with dye-labeled cell-lysate (from BL21 RIPL) in absence of sortase. (e) Overlay of distance distribution of two closest fluorescent spots portray an equivalent distribution centered at 0.60 ± 0.01 μm for (b) and 0.56 ± 0.01 μm for (c). (f) Schematics of the cleavage of hexa-histidine tag using TEV protease (g) TIRF image of Cdh23 protein immobilized on the surface. The signal was observed from the fluorescently labeled immunohistochemistry against the N-terminus 6xhis-tag. (h) TIRF image obtained after 3 h of incubation of the surface with TEV protease which cuts the 6xhis-tag significantly.

Our surface passivation comprised two stages of necessary cleaning with plasma and fresh piranha followed by silanization and pegylation (Fig. 1 in Ref. [13]). We executed silanization using APTES (3-Aminopropyl triethoxysilane) in acetone with 5% water that facilitates the formation of self-assembled monolayer by hydrolyzing the ethoxy groups in solution before impinging on the surface [14]. The RMS fluctuations in the height measured with an atomic force microscope (AFM) was 50.9 p.m., reflecting a large extent of homogeneity of the APTES layer (Fig. 2 in Ref. [13]). We cured the silanized surface at 110 °C to steer the reactive amine groups exposed for higher accessibility [15]. Using N-hydroxysuccinimide(-NHS) activation to carboxylate followed by nucleophilic attack, we subsequently attached a mixture (1:10) of bi-functional (maleimide-polyethylene-glycol-NHS, mal-PEG-NHS) and mono-functional PEG-NHS to surface attached amines (Fig. 1a). PEG, non-toxic and non-immunogenic, is known to impart resistance to nonspecific adsorption of biomolecules and cells. For single-molecule force spectroscopy (SMFS), PEG as freely jointed polymer serves as a spacer [16]. We used a thermodynamically bad solvent as pegylation buffer for minimizing the surface volume and thus, maximizing the grafting density desired for surface passivation [6] (Fig. 3 in Ref. [13]). The bi-functional mal-PEG-NHS having mal-groups exposed to the surface was harnessed for anchoring specific target proteins.

The density of mal-PEG-NHS ensured controlled density of immobilization, critical for single-molecule studies along with chip-based detections, microarrays (Fig. 1b and Fig. 4 in Ref. [13]). We optimized the density of mal-PEG-NHS to 10% by monitoring the ratio of unbinding events arising from single receptor-ligand interactions to multiple using SMFS.

### ESCORTing target proteins from whole cell-lysate

Using linear residue-specific enzyme reactions with bacterial transpeptidase sortase A (SrtA), we covalently attached the target proteins to the surface with specific orientations in a single step (Fig. 1a). SrtA present in *Staphylococcus Aureus*, *in vivo* catalyzes the transmembrane proteins containing enzyme-recognition motif, Leu-Pro-Xxx-Thr-Gly (LPXTG; Xxx is any amino acid), onto the cell-wall. The cysteinyl thiol group in SrtA nucleophilically attacks the threonine-glycine amide bond and forms a proteinyl-enzyme thioester intermediate (Fig. 1a). Another nucleophilic attack by a terminal oligoglycine present on the cell-wall releases the enzyme thereby tethering the protein to the cell-wall [17]. The high propensity of the thioester towards nucleophile from the amine group of polyglycine (kinetically most preferred for tetraglycine), makes sortagging extremely specific [18]. Adopting such sortagging, we modified two cell-adhesion cadherin class of proteins, cadherin 23 (Cdh23) and protocadherin 15 (Pcdh15) with the five residues of SrtA recognition site at the C-terminus (See Materials and Method). The two outermost extracellular domains (EC1+2) of Cdh23 and Pcdh15 are known to mediate heterophilic trans-interactions at the tip-inks of hair-cells [19]. It is well established that the short peptide chain containing as low as only five amino acid residues does not interfere with any measurements and ought to be serving as a linker to accommodate the possible rotational degree of freedom. Polyglycines those are pre-attached to surface through maleimide-cysteine Michael addition serve as a nucleophile to attack the thioester and engrafts proteins covalently onto surface (Fig. 1a).

To confirm the covalent bond as the only mode of association between protein and surface, we incubated Cdh23 and Pcdh15 in polyglycine attached surfaces in absence and presence of SrtA. Immunohistochemistry against N-terminus 6xHis-tag of the protein led to a TIRF signal only for SrtA mediated attachments, thereby ensuring specific covalent attachment of Cdh23 and Pcdh15 onto the surface (Figs. 9 and 10 in Ref. [13]).

**Fig. 2:**
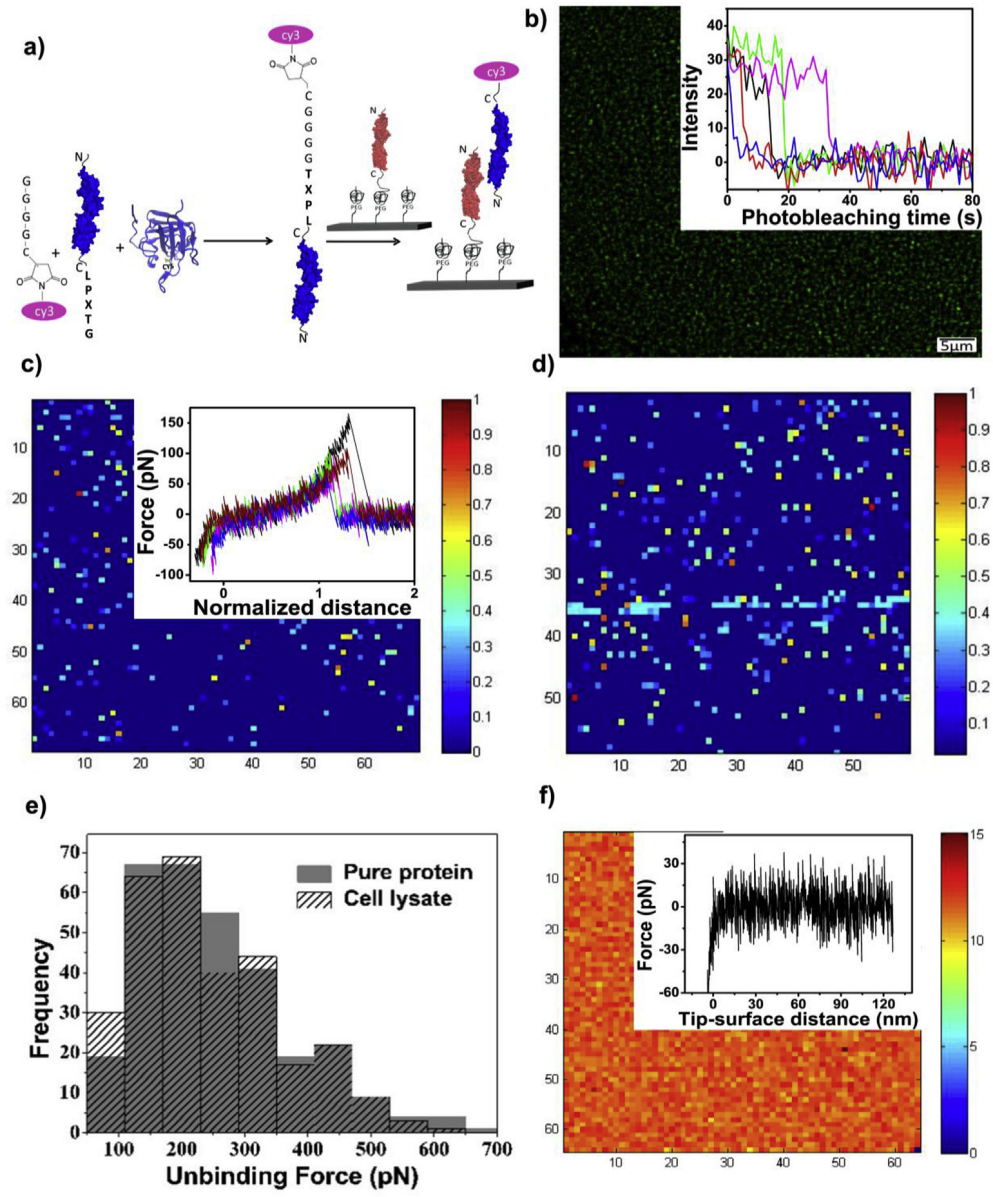
Pulling down of single protein-molecules directly from cell-lysate and performing force-spectroscopy on ESCORTed surfaces. (a) Scheme for sortagging of proteins with single fluorophore followed by pull-down. (b) Cy3-modified Pcdh15 from cell-lysate was pulled-down by the Cdh23 already ESCORTed on surfaces, at the single-molecule resolution (imaged under TIRFM) (Inset right: Single-step photobleaching of cy3 labeled pulled-down proteins). SMFM between cantilever ESCORTed with Pcdh15 and surface ESCORTed with Cdh23 from (c) affinity and chromatography pure-fraction (Inset right: Single molecule events characterized by PEG stretching and normalized by the tip-surface distance of the least binding force) and (d) crude cell-extract are shown by normalizing with the maximum force. (e) An overlay of the distribution of unbinding forces of interactions between Cdh23-Pcdh15, ESCORTed from pure fractions and cell-lysate show identical nature. (f) SMFM on control surfaces where coverslip was incubated with Cdh23 and cantilever was incubated with Pcdh15 from cell-lysate but in absence of sortase captures no events but only thermal-noise (Inset right: Force curves lacking the PEG stretch display absence of event).

To screen the high-specificity of sortagging and effective inertness of the surface, we compared surfaces decorated by ESCORTing proteins (Cdh23 or Pcdh15) from whole cell-lysate and from chromatography-purified fractions. We carried out immunohistochemical imaging for both surfaces using TIRF with primary anti-His antibody and Alexa Fluor 488 labeled secondary antibody against N-terminal 6xHis-tag (Fig. 1b, c and d). We observed comparable density of fluorophores along with identical distance distributions between closest fluorescent spots for both cell-lysate and purified proteins (Fig. 1e). To distinguish the specific fluorescent spots from nonspecific artifacts, we employed TEV protease to cut the N-terminal 6xHis-tag site [20] and observed >99% reduction in fluorescent signal (Fig. 1f, g and 1h) in three hours. TEV protease cleaves 6xHis-tag present in a protein. So incubation with TEV protease leads to cutting of 6xHis-tag, as a result antibody bound to 6xHis-tag will also get detached from the protein which led to decrease of fluorescence signal with time and at the end entire disappearance of it from the ESCORTed coverslip. To check whether some unwanted non-target protein is sticking on the surface we labeled whole cell extract from other cell lines randomly with Cy3 NHS ester as well as Cy-maleimide and incubated on the surface. We observed no fluorescence signal in these cases entailing that no non-target protein is sticking on the surface (Fig. 6 in Ref. [13]). No events on the same surface in SMFM, verified the specificity of the reaction as also the provable nature of surface to the contents of the cell extract (Fig. 2f).

### Functional studies of the ESCORTed proteins employing single molecule fluorescence microscopy and SMFM

It is the fundamental requirement for a surface to maintain the functionality of the proteins after immobilization. It is well known that Cdh23 and Pcdh15 interacts with each other in trans conformation through their N-termini to build tip-link connecting two neighboring stereocilia in inner ear hair cells [21] [19]. We probed the heterophilic interactions between these two proteins, Cdh23 and Pcdh15, to check the biocompatibility of the modified surfaces. To visualize such dimerization on surfaces, we carried out single-molecule pull-down of ligand-proteins (Cdh23 or Pcdh15) from whole cell-lysate. We first ESCORTed receptor-proteins (Cdh23 or Pcdh15) to surfaces. Ligand-proteins in cell-lysate were fused with single cy3 at their C-terminals by manifesting sortagging and allowed to interact with their receptors ESCORTed already on surfaces [24] (Fig. 2a). We imaged using TIRFM (Fig. 2b). Interactions arising from single molecules were identified from the single-step photobleaching (Fig. 2b). In control, no receptor protein was ESCORTed on the surface and surface was incubated only with labeled ligand protein. The absence of ESCORTed receptor-proteins on the surface did not attract ligands and showed no fluorescence signal in TIRF image (Fig. 12b in Ref. [13]).

This heterophilic interaction between Cdh23 and Pcdh15 is calcium dependent similar to other cadherin family proteins and removal of calcium leads to obliteration of this interaction which has previously been shown through analytical size exclusion chromatography [19]. Here, we employed the same concept to monitor the specificity of the surface. After pull down of labeled ligand protein from cell lysate on the surface using interacting receptor protein present already on the surface we incubated the surface in 1 mM EGTA (ethylene glycol-bis(β-aminoethyl ether)-N,N,N′,N′-tetraacetic acid) for 1 h. EGTA chelates out the calcium ions bound to proteins and facilitates the Cdh23-Pcdh15 unbinding. After one hour we imaged the surface using TIRFM and observed no fluorescence signal suggesting that no non-specific proteins from cell lysate was adhered to the surface (Fig. 5 in Ref. [13]). Corroborating to fluorescence results, SMFM also measured events as low as 0.4% for nonspecific incubation, nearly 10 times lower than specific and similar to Fig. 2f.

To check the extent and specificity of single molecule pull-down, we probed the unbinding force of protein-protein interactions at the single-molecule level using AFM. We ESCORTed purified Pcdh15 on the cantilever and Cdh23 to the glass-coverslip and subsequently quantified the strength of interaction at a constant pulling velocity of 5000 nms^-1^ (Fig. 2c). Next, we repeated the experiments by ESCORTing Pcdh15 from cell-lysate on the cantilever and Cdh23 from cell-lysate on the coverslip and measured the unbinding forces at same pulling velocity (Fig. 2d and e). We measured almost equal probability of single molecule interactions (5.3% and 5.4% respectively) between proteins both from cell lysate extract and purified proteins with similar force distribution (Inset of Fig. 2c and e). Only 2.7% of the total acceptable events showed multiple unbinding nature with proteins ESCORTed from cell-lysate and 3.2% for pure fraction (Fig. 14 in Ref. [13]). Both these fractions are very well within the Poisson distribution limit for single molecule detection [22].

To sum up, we have devised a protocol for effective surface passivation and ESCORTing of proteins that can be suitably deployed for single molecule studies. The exceedingly high passive nature of the surface and high specificity of the sortase reaction is appropriate for protein ESCORTing directly from cell-lysate, deploying the protocol substantial reduction in investment of time, energy and chemicals in purification. Sortase mediated reactions are strictly site-directed and hence, can be targeted for labeling any unstructured part in addition to C, N-terminals [18] [23]. The availability of both Ca^2+^-dependent and independent variants of sortase make the labeling more pliable to buffer [23]. Since sortagging is also reported on DNA [25] our protocol can be extended to ESCORT DNA as also to any cellular-organelle onto surfaces directly from cell-lysate.

## Materials and methods

### Passivation and functionalization of coverslips and cantilevers for covalent attachment of proteins employing sortase a click chemistry

The glass coverslips and Si_3_N_4_ cantilevers were first cleaned in a plasma chamber at low air pressure (typically 200–600 mtorr) and medium radio frequency radiation (∼10 MH) for 1 min at ambient temperature. Immediately after the plasma cleaning, the surfaces were immersed in a freshly prepared piranha solution (3:1 mixture of H_2_SO_4_ (Merck) and 30% (w/v) H_2_O_2_ (Merck)) for 2 h at room temperature. Cantilevers were placed in Piranha solution for 30 min followed by gentle washing in milliQ water. Piranha solution is very corrosive and should be treated with care. The coverslips were washed under flowing milliQ water and then sonicated for 5 min at 53 kHz. Coverslips used in single molecule force mapping using AFM were etched in 1 M KOH (Himedia) for 15 min. For fluorescence imaging, this step can, however, be avoided. Following the KOH treatment, the coverslips were washed rigorously and sonicated twice in milliQ water for 5 min each time at 53 kHz.

The functionalization of the coverslips for covalent attachment of target proteins employing the sortagging scheme involves 3 reaction steps as following:

(i) Silanization

Silanization with aminosilane is one of the most convenient methods of turning glass, quartz or silicon based surfaces reactive. We used 3-Aminopropyltriethoxysilane (APTES, Aldrich) in 5% of water in acetone and incubated the freshly cleaned coverslips and cantilevers in it for 30 min. Trace of water in APTES solution facilitates the formation of the silane monolayer prior to coming in contact with surfaces and avoids inhomogeneous multilayer formation. After incubation, the surfaces were washed several times with acetone (Merck) and water and subsequently dried in nitrogen environment. The coverslip and cantilevers were then placed at 110 °C for 1 h to increase the accessibility of the reactive amine groups.

(ii) Pegylation

Polyethylene glycol (PEG), non-toxic and non-immunogenic, is known for its applications against non-specific adsorption of biomolecules or cells onto surfaces. PEG with high molecular weight also serves as spacers to surface interactions. PEG solutions were prepared with methoxy PEG-succinimidyl valerate (PEG-SVA) (Laysan Bio) and maleidmide-PEG-SVA (Laysan Bio) in PEG buffer (100 mM NaHCO_3_, 600 mM K_2_SO_4_, pH 8.0) at a fixed concentration of 1 mg/μL. Depending on the demand of the application, the distance necessitated between the single molecules might vary. 10% (v/v) of maleimide-PEG-SVA in PEG-SVA was used for majority of the experiments. To appreciate the control over the distance between the single molecules, 1% (v/v) of maleimide-PEG-SVA in PEG-SVA was used for one experiment. Following pegylation, the coverslips and cantilevers were washed with milliQ water.

(iii) Introducing polyglycine on the surface to harness proteins via the sortase mechanism

The coverslips and cantilevers were incubated in 100 μM tetraglycine to N-terminus along with cysteine at C-terminus (GGGGC) (GenScript). The cysteine is for the addition of polyglycine to the maleimide group of surface attached PEG for 7 h at room temperature. The polyglycine stock was made in buffer A (50 mM HEPES, 50 mM NaCl, 25 mM KCl, 2 mM CaCl_2_, pH 7.5) along with 1 mM dithiothreitol (Sigma) to prevent disulphide bond formation. A large volume (50 μL) of polyglycine is used for the incubation of each coverslip to sequester all the free maleimide groups on the surface to prevent its reaction with the cysteinyl group in sortaseA or the protein to be tethered. The coverslips and cantilevers were washed with water and stored in vacuum until their use. For their most efficient use, the coverslips must be used within 5 days for an AFM experiment and can be used until 10 days for a TIRF experiment.

### Covalent, sortase assisted attachment of proteins onto the surface

200 nM of purified Cdh23 (and Pcdh15) and 240 nM of sortaseA were mixed such that the final volume was sufficient for the incubation of the coverslips (30 μL for each coverslip) and cantilevers (30 μL for each cantilever). Target protein to sortaseA concentration ratio was maintained to be 4:5. Buffer A was used for the reaction. Both coverslip and cantilever surfaces were incubated with the reaction mixture for 1 h at room temperature and subsequently washed gently with the buffer to remove the unreacted Cdh23 (or Pcdh15) and sortaseA.

In the case of attaching proteins directly from the cell lysate, the cell lysate was centrifuged at 13,000 rpm for 15 min and filtered using a 0.1μ filter. 10 μL of the cell lysate was incubated with 240 nM of sortaseA following the same protocol as above.

### Total internal reflection fluorescence (TIRF) imaging

The functionality as well as the surface passivity of the ESCORTed coverslips were imaged using an objective based inverted TIRF microscope (Olympus model No. IX3 combined with IX3 TIR MITICO TIRF illuminator) equipped with 488-nm, 532-nm and 633-nm diode laser systems for Alexa Fluor 488, Cy3 and Cy5 excitation, respectively. The fluorescence collected by an oil-immersion objective (60X, NA 1.49 Olympus) was splitted into two channels with a dichroic beam splitter and recorded using an electron-multiplying charge-coupled device (EMCCD) camera (Q Imaging Roller Thunder). The filters used were Quad band LF405/488/532/635-A-000 BrightLine Full Multi-Band Laser Filter set.

Proteins ESCORTed on coverslips were labeled with fluorophores in two different ways for imaging: immunohistochemically and enzymatically using sortase A. In immunohistochemical method, the 6xHis-tag present in the N-termini of both Cdh23 and Pcdh15 proteins were targeted. The coverslips after attaching proteins were incubated in 30 μL of 1:3000 dilution of monoclonal antibody against poly-histidine produced in mouse (Sigma) for 1 h at room temperature and subsequently incubated in PBS buffer (pH 7.4) for 5 min, 3 times to wash off the extra antibody adhered to the surface nonspecifically. Next, the coverslips were incubated in 30 μL of 2 μg/mL of Alexa Fluor 488 labeled goat anti-mouse (Invitrogen) for 1 h at room temperature. The coverslips were again similarly washed with the same PBS buffer and imaged under the total-internal-reflection fluorescence (TIRF) microscope. Imaging was done on number of different positions to cover the entire coverslip. The same was repeated for Cdh23 proteins (and Pcdh15 proteins) present in the cell lysate. In the enzymatic method, the C-terminus of the protein was decorated with single dye that helped in imaging of single molecule pull-down assay.

The fluorescence spots in the image were identified from the intensity maxima greater than a predetermined threshold and fitted to a two-dimensional Gaussian. The intensity threshold was kept as global constant for direct comparison between different surfaces. In case of single molecule, the single events were confirmed by single-step photobleaching of the fluorophore. After the identification of, the distance between the fluorescent spots were calculated and the shortest distances between two neighboring spots was plotted.

### Experiment employing TEV protease

After immobilizing proteins, the coverslip was incubated in 30 μL of 1:3000 dilution of monoclonal antibody against poly-histidine produced in mouse (Sigma) for 1 h at room temperature and subsequently washed with PBS buffer (pH 7.4), 3 times. Next, the coverslips were incubated in 30 μL of 2 μg/mL of 488 Alexa Fluor labeled goat anti-mouse (Invitrogen) for 1 h at room temperature. The coverslips were again similarly washed with the same PBS buffer and imaged under TIRF. Following imaging, the coverslip was incubated on 2.5 μM of TEV protease for 3 h at 30 °C. Images were taken every 5 min interval.

### Test for non-specificity and passiveness of the surface

The passive nature of the surface was verified by fluorescence imaging using TIRF microscope and single molecule force mapping using atomic force microscope.

**I. Surface Passivation**
  **(a)** The surface passivation against proteins were checked with 5 different cell lysates, namely, yeast cell extract, mammalian cell extract THP-1, HEK and RAW 264.7 and bacterial cell extract BL21 RIPL with Takara genes. Each of the cell lysates contained proteins of varying molecular weights.
  **(b)**The surface passivation against non-specific DNA was checked with 40-nt ss-DNA tagged with cy3 maleimide.

The cell lysates were centrifuged at 13,000 rpm for 15 min. Supernatants were collected, filtered using 0.1 μm filter (Millex 33 mm Durapore PVDF sterile) and used directly for reaction.

#### Fluorescence imaging using TIRF microscope

In order to check the non-specific attachments using fluorescence signal, the reactive amine groups in the proteins in cell lysate were labeled with cy3-NHS ester (GE Healthcare) prior to incubation with surfaces. The labeled cell lysate was washed with buffer A using a 3 kDa filter until the absorbance at 545 nm of the filtrate (λ_ex_ of cy3) is negligible. The absorbance of the supernatant at 545 nm was measured and the concentration of the dye attached to proteins was calculated to ensure labeling. The labeled cell lysate was incubated on the surface, in the absence of sortaseA, for 1 h and gently washed with buffer prior to imaging. The same was repeated for each of the cell lysates in addition to BL21 RIPL with Takara genes. For screening the non-specific attachment of DNA (Sigma) on the surface, 50 nM of cy3 maleimide (GE Healthcare) labeled 40-nt ss-DNA was incubated on the surface for 1 h. The coverslip was gently washed and imaged using TIRF. Imaging was done in several spots so as to cover the entire coverslip. In all these experiments, the total protein concentration was maintained >0.8 μM, much higher than required for studying protein-protein recognition of lower affinity.

#### Single molecule force mapping using atomic force microscope (AFM)

The cell lysate was incubated on the coverslip and cantilever, in the absence of sortaseA for 1 h and thereafter, gently washed with buffer. Single molecule force mapping experiment was performed using an AFM on several parts of the surfaces at a constant pulling velocity of 750 nm s^−1^. The scan area for each experiment was set to 640 nm * 640 nm and the step size was set according to the diameter of the cantilever tip (∼10 nm, gold coated Si_3_N_4_ with spring constant = 30 pN/nm). The stretching of any spacer, polyethylene glycol (PEG), either from cantilever or coverslips or both was chosen as positive events and the magnitude of their unbinding forces were plotted as intensity in Figures. In majority of the cases where cantilevers could not stretch any PEG, we plotted the thermal noise of the cantilever as intensity. The same was repeated for each of the cell lysate and the percentage of non-specific events was calculated for each of the cell lysates based on the positive events with respect to total.

**II.** Control experiments to check the extent of non-specific attachment of sortaseA onto the surface

The sortaseA was labeled with cy5 NHS (GE Healthcare) and incubated on the coverslip for 2 h to check their adherence to modified surfaces non-specifically.

**III.** Control experiments to check the extent of non-specific interaction of primary and secondary antibody

30 μL of primary antibody mouse anti-his (1:3000) was incubated on the PEGylated coverslip for 1 h at room temperature. The coverslip was then washed with PBS buffer, pH = 7.4, 3 times, 5 min like previous cases. Next, the coverslip was incubated in 30 μL of Alexa Fluor 488 labeled anti-mouse antibody from goat (2 μg/mL) for 1 h at room temperature and washed again similarly with the same PBS buffer and imaged under the TIFR microscope. Imaging was done in several parts to map the entire coverslip.

### Checking functionality/activity of proteins post ESCORTed to surfaces

#### Single molecule pull-down assay

Pull down of proteins from cell lysates was carried out to portray the heterophilic dimerization ability of the proteins ESCORTed on the surface. The pull down was monitored at the single molecule level by attaching single dye to specific counter proteins at their specific sites using sortagging. Alike ESCORTing, labeling target proteins with a single dye at specific site was also done in cell lysate in presence of other proteins without any purification. Pull-down mediated by heterophilic interactions were carried out by first ESCORTing Cdh23 on the PEG modified surface as described in the protocol above. Pcdh15 present in the cell lysate of BL21 RIPL with Takara genes was labeled in the C-terminus employing cy3 maleimide (GE Healthcare) tagged polyglycine using the same sortagging mechanism. The cell lysate containing the C-termini labeled Pcdh15 was incubated on the surface already modified with Cdh23 for 30 min. The coverslip was gently washed and imaged using TIRF to demonstrate the single molecule heterophilic pull down of Pcdh15.

#### Single molecule force spectroscopy and mapping to confirm high specificity of the surfaces

Single molecule force spectroscopy and mapping were performed for ESCORTed Cdh23 proteins from both purified stock and from a mixture in cell lysate of BL21 RIPL with Takara genes and compared. The scan area for each experiment was set to 24.75 μm * 24.75 μm with the step size of 330 nm. The AFM tip and coverslip were first brought into contact so that opposing proteins interacted. The tip was then withdrawn from the surface with a constant pulling velocity of 5000 nm s^−1^. Single molecule events were characterized from the freely-jointed stretching of the PEG polymer which anchored the protein onto the surface [16] [26]. The contour length of the stretched polymer was used to distinguish specific interactions from non-specific binding [27]. For mapping the force on image, the magnitude of the unbinding forces was plotted as intensity. In majority of the cases where cantilevers could not stretch any PEG, we plotted the thermal noise of the cantilever as intensity.

### AFM imaging of surface and calculation of root mean square deviation

AFM imaging was done in contact mode with cantilever (gold coated Si_3_N_4_ with spring constant = 10 pN/nm) after the amino functionalization and pegylation of the surface. The imaging was done on multiple parts of the surface on an area of 10 μm * 10 μm each.

### Expression and purification of sortaseA

The sortaseA (Δ59) in pET28a plasmid (pET28a-SrtAdelta59 was a gift from Hidde Ploegh (Addgene plasmid # 51138)) with N-terminal 6x-his tag was transformed into competent Ecoli BL21 (DE3) strain and plated onto Leuria-Bertini(LB)-kanamycin(Himedia) plates. The transformed cells were grown at 37 °C in LB media till OD_600_ reached approximately 0.5 and then induced with Isopropyl- β-d-thiogalactopyranoside IPTG (Himedia) to a final concentration of 0.5 mM. The cells were pelleted down after inducing the cultures at 25 °C for 16hr. These pellets were processed for purification of sortaseA. 5 mL of 50 mM HEPES (Himedia) buffer with 100 mM NaCl (Himedia), 50 mM KCl (Himedia), 2 mM CaCl_2_ (Himedia), pH 7.5 was added to re suspend the pellet after 3 freeze-thaw cycles. The bacterial cells were lysed under chilled conditions by using probe sonication working at 30% amplitude for 5 min with 15sec on/off pulse. The lysed cells were centrifuged at 4 °C for 20 min and the supernatant was loaded onto pre-equilibrated Ni-NTA resin for IMAC purification. Maximum amount of protein was eluted using 200 mM Imidazole (Himedia) using step elution process. The eluted protein fractions were further purified using size-exclusion chromatography with Superdex 75 16/30 column (Wipro-GE Healthcare). Purified protein was confirmed by analysing on SDS-PAGE. The protein was stored in buffer A containing 25 mM arginine and 25 mM glutamic acid.

### Expression and purification of Cdh23 and Pcdh15

Cdh23 and Pcdh15 constructs with N-terminal 6x-his tag were bought from GenScript and the sortaseA recognition site LPETG was genetically attached to the C terminus by Polymerase Chain Reaction (PCR). The cell adhesion proteins were expressed in BL21 RIPL and purified using affinity chromatography followed by size exclusion chromatography (SEC) with Superdex 75 16/30 column (Wipro-GE Healthcare) as mentioned elsewhere.

## Author contributions

G.S.S. and S.R. conceived the idea for the project. S.S. and J.P.H. performed the experiments. G.S.S. has contributed in the force-mapping using AFM. S.S. and S.R. prepared the manuscript. G.S.S. and D.D. expressed and purified Sortase A, Cdh23 and Pcdh15.

## Competing interests

Authors declare no competing financial interests.

## References

[1] Pawson T. (2003). Assembly of cell regulatory systems through protein interaction domains. Sci..

[2] Wilson D.S., Nock S. (2002). Functional protein microarrays. Curr. Opin. Chem. Biol..

[3] MacBeath G. (2002). Protein microarrays and proteomics. Nat. Genet..

[4] Evans E. (2001). Probing the relation between force — lifetime — and chemistry in single moelcular bonds. Annu. Rev. Biophys. Biomol. Struct..

[5] Hua B., Han K.Y., Zhou R., Kim H., Shi X., Abeysirigunawardena S.C., Jain A., Singh D., Aggarwal V., Woodson S. a, Ha T. (2014). An improved surface passivation method for single-molecule studies. Nat. Methods.

[6] Ha T., Rasnik I., Cheng W., Babcock H.P., Gauss G.H., Lohman T.M., Chu S. (2002). Initiation and re-initiation of DNA unwinding by the Escherichia coli Rep helicase. Nature.

[7] Heyes C.D., Groll J., Möller M., Nienhaus G.U. (2007). Synthesis, patterning and applications of star-shaped poly(ethylene glycol) biofunctionalized surfaces. Mol. Biosyst..

[8] Abad José M., Mertens Stijn F.L., Pita Marcos, Fernández Victor M., Schiffrin D.J. (2005). Functionalization of Thioctic Acid-capped Gold Nanoparticles for Specific Immobilization of Histidine-tagged Proteins.

[9] Reynolds N.P., Tucker J.D., Davison P.A., Timney J.A., Hunter C.N., Leggett G.J. (2009). Site-specific immobilization and micrometer and nanometer scale photopatterning of yellow fluorescent protein on glass surfaces. J. Am. Chem. Soc..

[10] Taniguchi Y., Kawakami M. (2010). Application of Halotag protein to covalent immobilization of recombinant proteins for single molecule force spectroscopy. Langmuir.

[11] Kindermann M., George N., Johnsson N., Johnsson K. (2003). Covalent and selective immobilization of fusion proteins. J. Am. Chem. Soc..

[12] Clow F., Fraser J.D., Proft T. (2008). Immobilization of proteins to biacore sensor chips using Staphylococcus aureus sortase A. Biotechnol. Lett..

[13] S. Srinivasan, J.P. Hazra, G.S. Singaraju, D. Deb, S. Rakshit, Data on surface modification for direct orientation-specific covalent attachment of proteins from cell-lysate for single molecule studies, DIB-D-17-00680.

[14] Aebersolds R.H., Teplow B., Hood L.E., Kent B.H. (1986). Electroblotting onto activated glass. J. Biol. Chem..

[15] Naik V.V., Städler R., Spencer N.D. (2014). Effect of leaving group on the structures of alkylsilane SAMs. Langmuir.

[16] Oesterhelt F., Rief M., Gaub H.E. (1999). Single molecule force spectroscopy by AFM indicates helical structure of poly ( ethylene-glycol ) in water. New J. Phys..

[17] Chalovich J.M., Eisenberg E. (2005). NIH public access. Biophys. Chem..

[18] Mao H., Hart S.A., Schink A., Pollok B.A. (2004). Sortase-mediated protein ligation: a new method for protein engineering. J. Am. Chem. Soc..

[19] Sotomayor M., Weihofen W. a, Gaudet R., Corey D.P. (2012). Structure of a force-conveying cadherin bond essential for inner-ear mechanotransduction. Nature.

[20] Parks T.D., Leuther K.K., Howard E.D., Johnston S.A., Dougherty W.G. (1994). Release of proteins and peptides from fusion proteins using a recombinant plant virus proteinase. Anal. Biochem..

[21] Kazmierczak P., Sakaguchi H., Tokita J., Wilson-Kubalek E.M., Milligan R. a, Müller U., Kachar B. (2007). Cadherin 23 and protocadherin 15 interact to form tip-link filaments in sensory hair cells. Nature.

[22] Stevens F., Lo Y.S., Harris J.M., Beebe T.P. (1999). Computer modeling of atomic force microscopy force measurements: comparisons of Poisson, histogram, and continuum methods. Langmuir.

[23] Hirakawa H., Ishikawa S., Nagamune T. (2012). Design of Ca 2+-independent Staphylococcus aureus sortase A mutants. Biotechnol. Bioeng..

[24] Madej M.P., Coia G., Williams C.C., Caine J.M., Pearce L.A., Attwood R., Bartone N.A., Dolezal O., Nisbet R.M., Nuttall S.D., Adams T.E. (2012). Engineering of an anti-epidermal growth factor receptor antibody to single chain format and labeling by sortase a-mediated protein ligation. Biotechnol. Bioeng..

[25] Koussa M.A., Halvorsen K., Ward A., Wong W.P. (2015). DNA nanoswitches: a quantitative platform for gel-based biomolecular interaction analysis. Nat. Methods.

[26] Smith S.B., Finzi L., Bustamante C., Bustamantet C. (1992). Direct mechanical measurements of the elasticity of single DNA molecules by using magnetic beads. Science.

[27] Zhang Y., Sivasankar S., Nelson W.J., Chu S. (2009). Resolving cadherin interactions and binding cooperativity at the single-molecule level. Proc. Natl. Acad. Sci. U. S. A..

